# Malnutrition and nutritional deficits as aggravating factors in Guillain-Barré Syndrome: a call for nutritional intervention in the Gaza Strip

**DOI:** 10.3389/fpubh.2025.1681063

**Published:** 2025-09-24

**Authors:** Abdel Hamid El Bilbeisi

**Affiliations:** ^1^Department of Clinical Nutrition, Faculty of Pharmacy, Al Azhar University of Gaza, Gaza Strip, Palestine; ^2^Department of Nutrition, School of Medicine and Health Sciences, University of Palestine, Gaza Strip, Palestine

**Keywords:** Gaza Strip, Guillain-Barré Syndrome, humanitarian crisis, malnutrition, nutritional deficiencies

## 1 Introduction

Guillain-Barré Syndrome (GBS) is a rare but serious autoimmune disorder affecting the nerves. It is often triggered by infections, especially those of the gut or respiratory tract (1). In mid-2025, the Palestinian Ministry of Health reported a sharp rise in GBS cases in the Gaza Strip (2). This increase highlights the worsening humanitarian crisis, widespread malnutrition, and greater vulnerability to neurological complications after infections (3). During June and July 2025, a total of 64 GBS cases were reported across Gaza governorates. The majority of cases, 46 (72%) were reported in July, while 18 (28%) cases were reported in June; with a male predominance of 69% (44 cases) occurring in males compared to 31% (20 cases) in females (2). Data on the number of GBS cases in Gaza, specifically from the Palestinian Ministry of Health (2), are drawn from internal surveillance reports that have not undergone peer review. While these reports provide critical real-time data, they should be interpreted with caution due to the lack of standardization in reporting protocols and potential underreporting.

Since March 2025, Gaza has been under an almost complete blockade, severely limiting access to vital food supplies, clean water, medical resources, and humanitarian aid (4). This blockade has triggered a dire nutritional emergency, with nearly 500,000 individuals experiencing famine-like conditions and a significant surge in acute malnutrition rates, particularly affecting children under 5 years old and pregnant or breastfeeding women (4). Simultaneously, the breakdown of water, sanitation, and hygiene (WASH) systems has contributed to a sharp rise in diarrheal illnesses and heightened infection risks (5). Nutritional deficiencies, including micronutrients shortages and chronic anemia, compromise immune function, hinder nerve regeneration, and intensify both the likelihood and severity of GBS (6). This paper investigates the exacerbating impact of nutritional inadequacies on GBS occurrence and clinical outcomes in Gaza, calling for urgent, coordinated nutritional and medical responses amid the ongoing humanitarian emergency. While submitted as an Opinion Article, this piece adopts a hybrid structure, grounded in clinical observations and supported by emerging data from humanitarian field reports, local surveillance, and global literature. The goal is not to present a systematic review, but rather to offer an evidence-informed expert commentary that situates the current neurological health crisis within a broader context of malnutrition, infectious disease burden, and health system collapse. This approach aims to inform policy, advocacy, and clinical response in acute conflict zones like the Gaza Strip.

## 2 Discussion

### 2.1 Gaza's malnutrition and humanitarian crisis

Since the near-total blockade of Gaza beginning on March 2, 2025, the region has been thrust into a severe humanitarian crisis, with critical shortages of food, water, medical supplies, and humanitarian assistance (4). This situation has resulted in widespread acute food insecurity, with nearly 500,000 people experiencing famine-like conditions, classified under IPC Phase 5 (4). By July 2025, the World Health Organization reported alarming figures: one in five children under five in Gaza was suffering from acute malnutrition, marking a 4 fold increase in just 2 months (7). Projections from UNICEF and World Food Program (WFP) estimate that around 71,000 children and 17,000 pregnant or breastfeeding women will need urgent nutritional support through the coming year (8). Médecins du Monde also reported that by November 2024, the prevalence of acute malnutrition had reached 17% among infants and 19% among pregnant or breastfeeding women, a stark contrast to pre-conflict rates of less than 1%. In some areas, severe acute malnutrition affected up to 25% of children and mothers by spring 2025 (9). The breakdown of Gaza's water, sanitation, and hygiene (WASH) infrastructure has compounded the crisis, leading to a dramatic rise in diarrheal diseases, which now affect 90% of children under five (5). This combination of malnutrition, disease, and infrastructure collapse has created an environment ripe for the emergence and exacerbation of infectious and post-infectious diseases, including GBS (1).

### 2.2 Surge of Guillain-Barré Syndrome (GBS) and plausible drivers

A significant surge in GBS and Acute Flaccid Paralysis (AFP) cases has been observed across Gaza since mid-2025, although comprehensive surveillance data is lacking. Local healthcare workers have reported an unusual rise in these conditions, which disproportionately affect both children and adults (2). The spike in GBS cases appears to be linked to increased enteric and respiratory infections in the region, exacerbated by the ongoing food and water shortages, as well as poor sanitation (3). The main infectious agents suspected to play a role include *Campylobacter jejuni* and *enteroviruses*, which are known to trigger GBS, particularly in immunocompromised individuals. The prevailing malnutrition crisis, with widespread deficiencies in key micronutrients, likely amplifies the vulnerability to these infections and their subsequent neurological effects.

### 2.3 Role of nutritional deficiencies and immune impairment

Malnutrition, especially micronutrient deficiencies, severely weakens the immune system. Lack of iron, folate, vitamin B12, and zinc reduces the body's ability to fight infections and repair nerves (10). In Gaza, more than 15% of children under two suffer from acute malnutrition and wasting (11). These deficiencies damage immune defenses and mucosal barriers. As a result, people become more vulnerable to infections like *Campylobacter jejuni* and *enteroviruses*. These infections are the main triggers of GBS in low-resource areas (12). Furthermore, malnourished individuals exhibit reduced leukocyte activity, compromised gut integrity, and diminished capacity for nerve repair, all of which elevate the risk of severe, post-infectious neurological complications such as GBS (13). The convergence of these factors forms a perfect storm for the amplification of GBS cases in Gaza, necessitating a multisectoral response to address the underlying nutritional and healthcare challenges.

Table 1 summarizes key epidemiological and nutritional data from Gaza during June–July 2025. The surge in GBS cases coincides with alarming rates of acute malnutrition among children and pregnant women, as well as significant strain on healthcare resources, including ICU admissions and ventilator needs.

**Table 1:** Key statistics on Guillain-Barré Syndrome and malnutrition in Gaza (June–July 2025).

**Indicator**	**Value**	**Source/notes**
Total GBS cases reported (June–July 2025).	64 cases	Palestinian Ministry of Health (2)
Percentage of GBS cases in July.	72% (46 cases)	Palestinian Ministry of Health (2)
Male predominance in GBS cases.	69% (44 cases)	Palestinian Ministry of Health (2)
Acute malnutrition rate in children under 5.	20%	WHO report (7)
Acute malnutrition in pregnant/breastfeeding women.	19%	Médecins du Monde (9)
Children under two with acute malnutrition/wasting.	>15%	Albelbeisi et al. (11)
Children under five with diarrheal diseases.	90%	Abuzerr (5)
Percentage of GBS/AFP patients needing ICU care.	~30%	Palestinian Ministry of Health (2)
Percentage of ICU GBS patients requiring ventilation.	>85%	Palestinian Ministry of Health (2)
People facing famine-like conditions.	~ 500,000	OCHA (4)
Children needing urgent nutritional support.	71,000	UNICEF/WFP (8)
Pregnant/breastfeeding women needing support.	17,000	UNICEF/WFP (8)

### 2.4 Clinical severity and health system constraints

The clinical severity of GBS in Gaza has been striking, with preliminary reports indicating that up to 30% of GBS or AFP patients required intensive care unit (ICU) admission, and over 85% of those needed mechanical ventilation due to respiratory failure (2). This suggests that the majority of GBS cases in Gaza present with severe neurological involvement. The outcome of many patients, particularly children, has been poor due to delayed access to care, inadequate healthcare infrastructure, and the absence of essential therapies such as intravenous immunoglobulin (IVIG) or plasmapheresis (14). The malnutrition epidemic exacerbates the severity of GBS, as patients with low nutritional reserves are more likely to experience complications, longer recovery times, and even death due to the lack of intensive care support (6). Additionally, the healthcare system in Gaza is under severe strain, with critical shortages in ICU beds, ventilators, and medications, further hindering the ability to provide optimal care to GBS patients.

### 2.5 Recommendations and integrated response

An urgent, coordinated short-term response is needed to reduce the surge of GBS in Gaza. Surveillance should be expanded to include adults and integrate malnutrition assessments during GBS investigations. Diagnostic efforts must prioritize detecting pathogens like *Campylobacter jejuni* and *enteroviruses*, supported by strengthened laboratory capacity for cerebrospinal fluid analysis, electrophysiological studies, and Polymerase Chain Reaction (PCR) testing. Clinical care improvements are critical, particularly increasing ICU capacity for mechanical ventilation and respiratory support, alongside securing essential treatments such as intravenous immunoglobulin (IVIG) and plasmapheresis, especially for severely malnourished patients. Immediate nutritional interventions must also be scaled up, including supplementary feeding programs targeting children, pregnant women, and breastfeeding mothers, and distributing therapeutic foods and lipid-based nutrient supplements to vulnerable groups.

For long-term sustainability, efforts should focus on rebuilding Gaza's health system infrastructure and restoring water, sanitation, and hygiene (WASH) services to reduce infectious disease transmission. Rehabilitating WASH infrastructure, particularly water chlorination and sanitation improvements in overcrowded shelters, is essential. Additionally, integrated multisectoral programs combining nutrition, infection control, and standardized surveillance systems will strengthen resilience against future outbreaks. Community engagement and education remain vital, with Non-Governmental Organizations (NGOs) and local leaders promoting awareness of GBS, its causes, and the importance of early medical care, as well as reinforcing nutrition and hygiene practices to improve overall health outcomes.

## 3 Limitations

This commentary faces key limitations due to the conflict setting in Gaza, including reliance on unverified internal surveillance and anecdotal clinical reports, which may involve diagnostic inconsistencies and underreporting. The blockade and security constraints hinder independent data collection and verification, limiting accurate assessment of GBS incidence and its link to malnutrition. Additionally, the lack of baseline pre-conflict health data restricts comparisons and weakens causal inferences. Despite these constraints, the convergence of emerging field data, clinical patterns, and well-established biological plausibility provides a compelling foundation for the urgent recommendations proposed. Future investigations should prioritize standardized surveillance systems, expanded diagnostics, and access to population health metrics to strengthen evidence-based responses in humanitarian crises.

## 4 Conclusion

The GBS surge in Gaza is not an isolated neurological event, it is a sentinel indicator of catastrophic nutritional collapse, public health breakdown, and infectious diseases proliferation. Malnutrition, anemia, and micronutrients deficits have likely magnified both vulnerability to GBS-triggering infections and severity of disease. Urgent, sustained, and integrated action is required, linking nutrition restoration, infection control, clinical capacity building, and humanitarian access to prevent further avoidable morbidity and mortality. Additionally, this commentary presents a novel perspective by linking malnutrition and micronutrient deficiencies to the worsening of GBS during humanitarian crises. It emphasizes the underexplored intersection between nutritional collapse and post-infectious neurological disorders, using the Gaza Strip as a case study. The manuscript introduces an integrated framework that highlights the need for nutritional interventions as part of neurological and infectious disease responses in conflict-affected settings.
